# Expanded endoscopic endonasal transpterygoid transmaxillary approach for a giant trigeminal schwannoma

**DOI:** 10.3171/2020.4.FocusVid.19904

**Published:** 2020-04-01

**Authors:** Rafael Martínez-Pérez, Marcus Zachariah, Ruychen Li, Giuliano Silveira-Bertazzo, Ricardo L. Carrau, Daniel M. Prevedello

**Affiliations:** Departments of 1 Neurosurgery and; 2 Head and Neck Surgery, Wexner Medical Center, The Ohio State University, Columbus, Ohio

**Keywords:** schwannoma, skull base, endonasal, infratemporal, minimally invasive, video

## Abstract

Atypical trigeminal schwannomas (ATSs) are notorious for their ability to invade the skull base. An expanded endoscopic endonasal approach (eEEA) provides direct access to the tumor with no need for cerebral retraction or manipulation of neurovascular structures. Herein, we present a case of a large temporal fossa extradural lesion with secondary invasion of the sella, clivus, and temporal and infratemporal fossae in a 49-year-old male with severe vision loss. A transpterygoid transmaxillary approach was performed. Gross-total removal was achieved and pathology revealed the diagnosis of ATS. Visual function fully recovered in the right side and the patient has been uneventfully followed since surgery.

The video can be found here: https://youtu.be/6pSwdYsN9hk.

## Transcript

### 0:21 Case presentation

This video represents an expanded endonasal transpterygoid transmaxillary approach for resection of a giant trigeminal schwannoma.

0:30 Patient was a 49-year-old gentleman who presented with 1-year-history of vision loss on left side and progressive deterioration of his vision on the right side. He also had numbness on the left side of the face.

0:43 Head CT and an MRI showed the large lesion occupying the sphenoid, the clivus, the temporal fossa, as well as the infratemporal fossa, with expansion into the nasal cavity and proptosis.

0:58 We initially considered the preauricular infratemporal access to the tumor, but we decided to perform an expanded endonasal approach in order to reach all compartments of the lesion and optimize complete resection of the tumor. We explain to the patient that in case a total resection is not achieved we could complement with a transcranial approach.

### 1:24 Discussion on anatomical landmarks

We then mapped the tumor in relation to the landmarks of the skull base, the middle cranial fossa component of the tumor is located above the petrous internal carotid artery, and in the infratemporal component of the tumor, located below the petrous carotid artery.

### 1:39 Left middle turbinectomy

We then defined those landmarks, and proceeded to perform the surgery. An EEA was started regularly initially through the left nostril and a middle turbinectomy was accomplished. We exposed the bulla ethomoidalis as well as the uncinate process.

### 1:58 Left ethmoidectomy and uncinectomy

We removed the bulla ethmoidalis in order to access the maxillary sinus. We removed the uncinate process, performed a left-side uncinectomy, accessing then the maxillary sinus.

### 2:15 Medial maxillectomy

We could see the maxillary periosteum and bone that was expanded inside the maxilla. An inferior turbinectomy was accomplished to complete a medial maxillectomy for this patient. That maneuver allowed us to observe and visualize the inferior aspect of the tumor related to the infratemporal fossa later on in the case.

2:41 The mucosa was elevated and we were able to get control of the sphenopalatine vessel coming out the sphenopalatine fossa. The visualization of the tumor bulging on the posterior aspect of the maxilla was completed.

### 2:56 Left anterior maxillectomy

We decided to perform an anterior maxillectomy. We performed an incision at the mucosa medial to the pyriform aperture. The mucosa was reflected medially and the bone of the pyriform area was exposed lateralizing the rest of the mucosa, exposing the anterior wall of the maxilla.

3:20 We drilled the anterior wall of the maxilla, still through the nostril on the left side, and we were able to access the anterior aspect of the antrum of the maxillary sinus.

### 3:31 Harvesting of the right nasoseptal flap

We then moved our dissection to perform a nasoseptal flap on the right side. The septum was exposed, the flap was reflected posteriorly, and we drilled the bone of the septum in order to expose the contralateral mucosa of the sinonasal cavity.

3:54 A reverse flap was then created, and the denuded portion of the flap septum anteriorly was accomplished. With a complete medial maxillectomy, we were able to expose the entire ventral aspect of the bulging perspective of the tumor.

4:16 We then removed the mucosa in the posterior wall of the maxilla to allow lateral retraction of the pterygopalatine fossa.

### 4:21 Tumor debulking

We then removed the tumor by debulking the tumor located on the sphenoid sinus initially. And then, we decided to perform an extracapsular of the tumor. So, after debulking we were able to go around the tumor and surround the dissection. Dissecting the tumor away from the planum sphenoidale, as you can see in the image, as the bone was completely eroded.

4:55 We were able then to dissect and remove the tumor away from the dura at the anterior skull base. As we dissected more posteriorly, we were able identify that on the left side the tumor was really invasive to the structures. We removed the entire component of the tumor located on the sphenoid, as well as on the clivus area, exposing the carotid on both sides. The carotid was dehiscent on the ride and left sides of the paraclival region.

5:36 We observed both carotid arteries. You can see, in correlation with the anatomy in this picture, the location of the landmarks. Doppler was essential for the safety resection in this case. Once the midline component of the tumor was resected, we moved to the lateral component, behind the pterygopalatine fossa.

6:02 We dissected the tumor away from the lateral aspect of the planum sphenoidale and the ventral aspect of the cavernous sinus.

### 6:16 Resection of the posterolateral component of the tumor

We decided not to go through the pterygopalatine fossa directly, but we used a 45° endoscope behind the pterygopalatine fossa, in order to preserve its content.

6:27 We removed the tumor more lateral to allow more visualization of the pterygopalatine fossa, as you can see in this portion of the video. With angled scopes, behind the pterygopalatine fossa, we were able to go all the way to the dura of the middle temporal fossa, as you can see pulsating in this region after resection of the tumor.

6:50 That area was confirmed by image guidance expanding inferiorly as the pressure caused by the tumor was released. We then followed the dissection of the tumor from the cavernous sinus, all the way laterally, in order to accomplish as complete resection as possible.

7:12 Immediately laterally to the internal carotid artery, in the superior orbital fissure, gently resection of the tumor in this region has been accomplished. Immediately behind the pterygopalatine fossa, we removed the capsule of the tumor that was present in the infratemporal fossa. The infratemporal fossa herniated superiorly as you can see also in this image.

7:44 We were able, with image guidance, to confirm that we were past the position of the tumor. Now, at the middle cranial fossa, once again, we confirmed the brain herniated back into its regular position.

7:58 Navigation was essential for us to confirm the complete resection of the tumor. All the components of the tumor were resected, including the sphenoid, clival, middle cranial fossa, and infratemporal fossa components.

### 8:16 Skull base reconstruction using nasoseptal flap

The anterior maxillectomy was very important for us to pass instruments in parallel and use two corridors to approach this large lesion.

8:26 We then covered both carotid arteries with the nasoseptal flap that was harvested in the beginning of the case.

### 8:32 Postoperative results

MRI showed the complete resection of the tumor with no signs of complications, and reestablishment of the position of the temporal lobe on the left side.

8:43 Patient improved his vision to normal function in the right side with improvement of his numbness of the left side of the face, and he persisted amaurotic in the left side.

8:52 Pathology showed an atypical schwannoma with a Ki-67 of 30%.
